# Biomotors, viral assembly, and RNA nanobiotechnology: Current achievements and future directions

**DOI:** 10.1016/j.csbj.2022.11.007

**Published:** 2022-11-11

**Authors:** Lewis Rolband, Damian Beasock, Yang Wang, Yao-Gen Shu, Jonathan D. Dinman, Tamar Schlick, Yaoqi Zhou, Jeffrey S. Kieft, Shi-Jie Chen, Giovanni Bussi, Abdelghani Oukhaled, Xingfa Gao, Petr Šulc, Daniel Binzel, Abhjeet S. Bhullar, Chenxi Liang, Peixuan Guo, Kirill A. Afonin

**Affiliations:** a University of North Carolina at Charlotte, Charlotte, NC 28223, USA; b Wenzhou Institute, University of China Academy of Sciences, 1st, Jinlian Road, Longwan District, Wenzhou, Zhjiang 325001, China; c University of Maryland, College Park, MD 20742, USA; d New York University, Department of Chemistry and Courant Institute of Mathematical Sciences, Simons Center for Computational Physical Chemistry, New York, NY 10012, USA; e Institute for Systems and Physical Biology, Shenzhen Bay Laboratory, Shenzhen, Guangdong 518107, China; f University of Colorado Anschutz Medical Campus, Aurora, CO 80045, USA; g University of Missouri at Columbia, Columbia, MO 65211, USA; h Scuola Internazionale Superiore di Studi Avanzati, via Bonomea 265, 34136 Trieste, Italy; i CY Cergy Paris Université, CNRS, LAMBE, Cergy, France; j National Center for Nanoscience and Technology of China, Beijing 100190, China; k Arizona State University, Tempe, AZ, USA; l The Ohio State University, Columbus, OH 43210, USA

**Keywords:** ISRNN, RNA nanotechnology, Biomotors, Therapies, Drug delivery, Virus assembly

## Abstract

The International Society of RNA Nanotechnology and Nanomedicine (ISRNN) serves to further the development of a wide variety of functional nucleic acids and other related nanotechnology platforms. To aid in the dissemination of the most recent advancements, a biennial discussion focused on biomotors, viral assembly, and RNA nanobiotechnology has been established where international experts in interdisciplinary fields such as structural biology, biophysical chemistry, nanotechnology, cell and cancer biology, and pharmacology share their latest accomplishments and future perspectives. The results summarized here highlight advancements in our understanding of viral biology and the structure–function relationship of frame-shifting elements in genomic viral RNA, improvements in the predictions of SHAPE analysis of 3D RNA structures, and the understanding of dynamic RNA structures through a variety of experimental and computational means. Additionally, recent advances in the drug delivery, vaccine design, nanopore technologies, biomotor and biomachine development, DNA packaging, RNA nanotechnology, and drug delivery are included in this critical review. We emphasize some of the novel accomplishments, major discussion topics, and present current challenges and perspectives of these emerging fields.

## 1 Introduction

Nucleic acid engineering has been gaining considerable momentum for over a decade since the first meeting of the International Society of RNA Nanotechnology and Nanomedicine (ISRNN). This massive research effort is inspired by the immense potential that RNA offers to basic research and in clinical settings. RNA is a natural biopolymer that is highly integrated in a plethora of mechanisms essential for life and thus, understanding RNA’s folding, structure, and functions would allow for the rational design of RNA-based therapies and nanodevices suitable for biomedicines [1], [2]. As the translational aspects of the field have advanced, there have been new RNA therapies approved for clinical use [3], most notably-two SARS-CoV-2 mRNA vaccines that were rapidly developed and introduced during the COVID-19 pandemic. Despite these successes, the numerous translational challenges of RNA therapeutics [4], [5], [6], [7], [8] still preclude their broader biomedical applications. With the increasing number of research teams whose work focuses on overcoming these barriers, the emerging RNA field keeps moving towards curative goals.

In order to advance the applications of RNA in the fields of nanotechnology and nanomedicine, an interdisciplinary effort must be undertaken. To further this goal, the ISRNN has hosted a biennial meeting series, entitled “Biomotors, Viral Assembly, and RNA Nanobiotechnology”. At these events, researchers gather from across the globe to share and discuss their findings in each of these areas, with the most recent boasting presentations from over 15 countries by 68 independent researchers. This critical review aims to cover recent research highlights and perspectives in selected topics; namely, we discuss advancements in the fields of biomotors and biomachines, nanopores, nanozymes, viral frameshifting elements, RNA structure prediction and assessment, and RNA nanotechnology.

## 2 Research highlights

### 2.1 Biomotors and biomachines

2.1

#### 2.1.1 The push-to-open mechanism and the challenge of tethered mechano-sensitive ion channel NompC

**Fig. 1:**
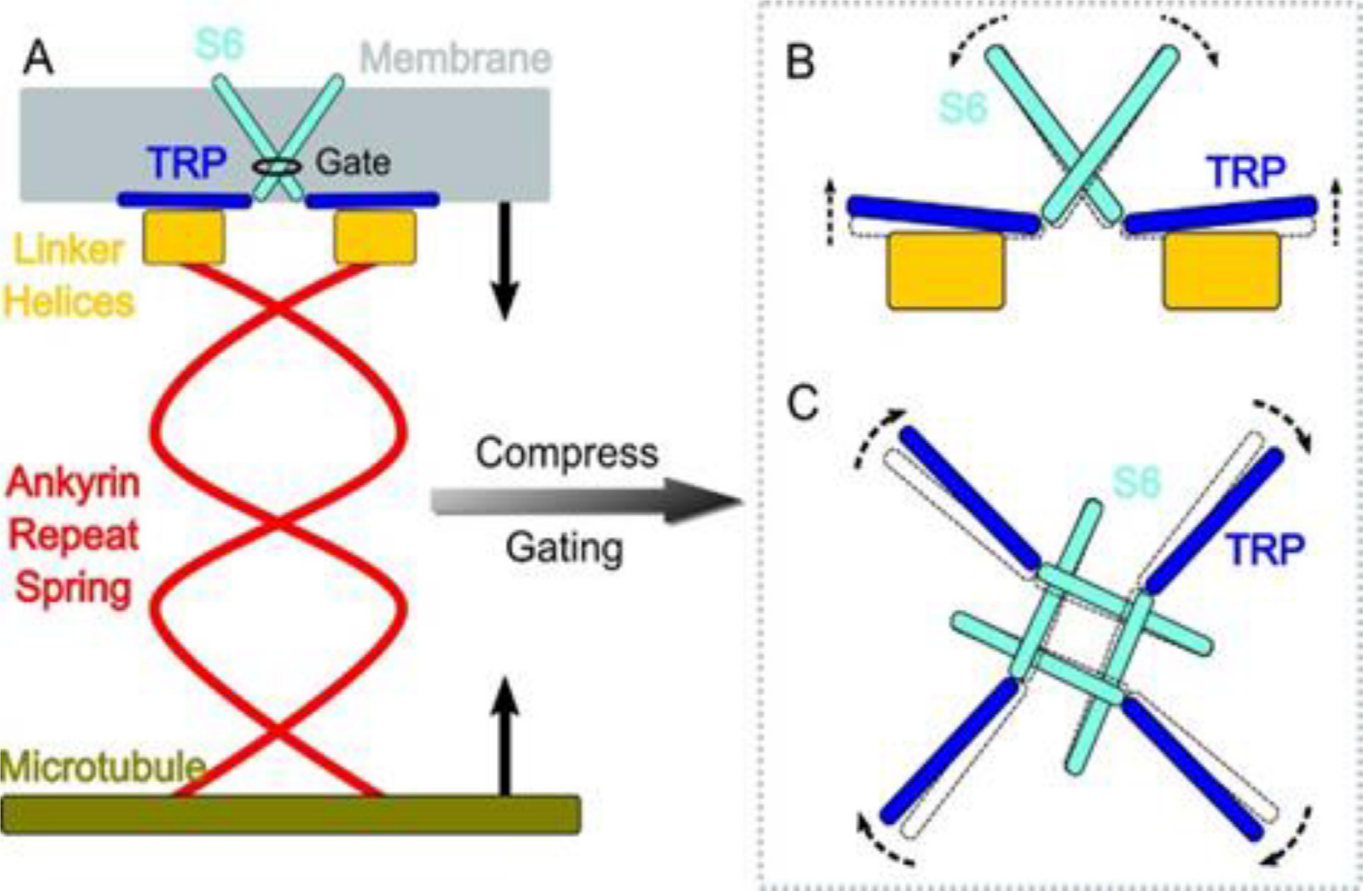
(A) The compression of the ankyrin repeat (AR) region will generate a pushing force and a torque on the linker helix (LH) domain, pointing to the extracellular side. (B) The LH domain further pushes the transient receptor potential (TRP) domain, leading to a tilt (side view), and (C) a clockwise rotation of the TRP domain (looking from the intracellular side). The motion of the TRP domain pulls the S6 helices to slightly tilt and rotate, which dilates the constriction site of the pore.

The 2021 Nobel Prize in Physiology and Medicine was given to Professors David Julis and Aderm Patapoutian for their discoveries of receptors that sense heat and pain. These breakthroughs launched intense research activities that lead to a rapid increase in our understanding of how our nervous system senses changes in temperature and mechanical stimuli. NompC is a TRP (Transient Receptor Potential) channel that is in the same family as the capsaicin receptor TRPV1. NompC is also a special mechanosensitive ion channel responsible for the sensation of touch and balance in Drosophila melanogaster [9]. Based on a cryo-EM structure of NompC [10], the team performed all-atom molecular dynamics simulations and electrophysiological experiments to study the atomistic details of NompC gating. As shown in Fig. 1, work done in the Shu group showed that NompC could be opened by compression of the intracellular ankyrin repeat domain but not by stretching, and that a number of hydrogen bonds along the force conveyance pathway are important for the mechanosensitivity [11]. Under intracellular compression, the bundled ankyrin repeat region acts like a spring with a spring constant of ∼13 pN/nm and transfers forces at a rate of ∼1.8 nm/ps. The linker helix region acts as a bridge between the ankyrin repeats and the transient receptor potential (TRP) domain, which passes on the pushing force to the TRP domain to undergo a clockwise rotation, resulting in the opening of the channel. This could be the universal gating mechanism of similar tethered mechanosensitive TRP channels, which enable cells to feel compression and shrinkage.

The Shu group’s experimental collaborators, Prof. Zhiqiang Yan’s group, found, using the cell-attached patch clamp method that positive pressure acted on the NompC expressed by Drosophila melanogaster S2 neuro cells, which simulated the compression of the gating spring on NompC [11]. From this experiment, electrical signals were detected from the cells, which indicated that the NompC channel was open in response to the compression of gating spring. By using the cell-attached method with negative pressure, there was no obvious current detected. On the other hand, the outside-out patch clamp method with negative pressure which also simulate the compression of gating spring, they can detect obvious current. These experiments validated the Shu group’s findings from MD simulations using *in vitro* methods. These findings were further validated by Prof. Michael Krieg’s research group via an in vivo *C. elegans* behavior experiment [12]. They found that the movement of *C. elegans* was controlled by two ion channels. Straight-forward movement was controlled by the TREK2 channel, a mechanosensitive potassium channel that can sense membrane stretching and transmit electric signals. The omega turn movement of *C. elegans* was found to be controlled by the NompC channel, which is stimulated by cellular compression [11]. NompC-knock out *C. elegans* can only go straight while the TREK2-knocked out C elegans can only perform the omega turn movement. Therefore, they summarize an interesting physical model of *C. elegans*. When the *C. elegans* moves straight, the TREK2 channel opens due to the stretch of nerve cells while NompC remain closed. On the contrary, when s *C. elegans* performs an omega turn, the sensory cells are compressed, opening the NompC channel while TREK2 is closed. These findings showed that NompC plays key roles in sensing touch and proprioception in *C. elegans*, which will provide promising logic element for designing smart *C. elegans*.

The push-to-open gating mechanism of NompC represents a special mechanism for sensing the compression of sensory cells, which is different from other traditional mechano-sensitive ion channels that mainly rely on membrane surface extension and respond to cell expansion. This model provides a promising area for designing smart biosensors in response to mechanical stimuli. Although the sequence and structure of other TRP ion channel family members, such as TRPV1 and TRPV4, is similar to NompC, whether the gating mechanism of these TRP channels obeys the push to open model remains an open challenge for structural biology. In the near future, the humans will begin to expand their explorations of space. The low gravity conditions in space pose a large challenge to the health of astronauts. Astronauts will face quite different health challenges from those living on earth. The relative studies on the gating of TRP channels under extreme conditions such as low gravity will give new insight to solve these problems.

#### 2.1.2 The asymmetrical revolving motor mechanism

**Fig. 2:**
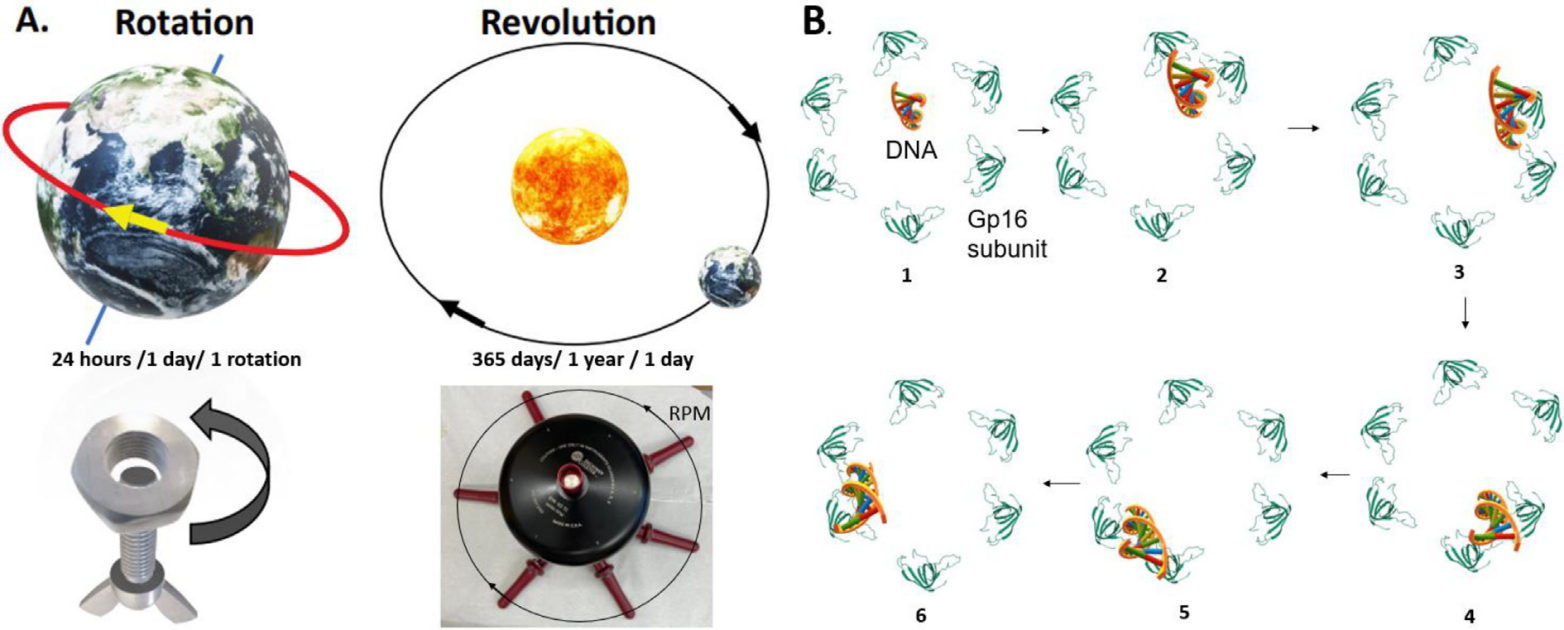
Differentiation of revolving mechanism from the rotation mechanism. Rotation is the object turning along its own axis. Revolving is one object moving around a second object. (A) Earth rotates one round every 24 h, analogous to the rotation of the bolt and nut (B). (C) Earth moving around the sun every 365 days per circles. Revolution is analogous to the RPM (revolution per minute) counting of rotors in ultracentrifugation (D). (E) The DNA translocation motor used a revolving mechanism. The revolving is a result of the sequential action of individual subunits trafficking DNA. DNA hugs the channel wall via sequential handoffs via dimerization of individual gp16 subunits (PDB 2KCA) [23]. This sequential dimerization of the gp16 subunits is ATP driven and traffics one strand of the DNA through the connector through touch and go interactions.

A key characteristic of “living beings” is movement. These motion activities are usually performed by ATPase motors [13], [14]. One of the most important motor functions is dsDNA translocation, which occurs during DNA replication, cellular mitosis, plasmid conjugation, DNA repair, and dsDNA viral genome packaging. The motor of these essential living processes shares plenty of similarities, such as the structure and mechanism. A rotatory mechanism has become a common belief due to the helical structure of the substrate DNA; however, the rotation mechanism is questioned based on several experimental data [15], [16]. To solve this problem, a revolving mechanism is proposed [17]. Rotation involves the turning of an object around its own axis like Earth moving around its axis every 24 h, while revolving is the circular motion of an object around a secondary object, similar to Earth orbiting the sun every 365 days. Studies of the structure of biological motors in different species have suggested a similar revolving mechanism that can transport the long dsDNA genome. This mechanism is established in the dsDNA packaging motor of the bacterial virus phi29, which uses six ATPases to work in sequential action (Fig. 2) [18], [19]. Several other biomotors have been reported to function in the revolving mechanism as well, such as the packaging motor of herpesviruses [20], the cell division motor of Escherichia coli FtsK [21], and plasmid conjugation motor TraB in streptomyces [22]. The studies of these motors suggest an asymmetric hexameric structure for transporting dsDNA in the sequential mechanism of action that is the motor contains four monomer subunits and one dimeric subunit arranged in an asymmetrical structure. The finding may help understand why huge genomes, including chromosomes, translocate in complex systems without coiling and tangling, which will decrease the speed of dsDNA translocation and consume more energy.

### 2.2 Nanopore and nanozyme technologies

#### 2.2.1 Protein fingerprinting through nanopores

Nanopore technology has demonstrated its ability to detect fine molecular details, allowing the study of subtle changes in protein or RNA conformation [24], [25], characterization of short polypeptide chains with single amino acid resolution, detect and identify specific amino acids [26], [27] and detect subtle chemical modifications [28], [29] or post-translational modifications [30], [31] all which led to the hope of directly sequencing whole proteins. In addition, the biological nanopore can be reengineered to be inserted in lipid bilayer system or commercially available platform [32], [33].

**Fig. 3:**
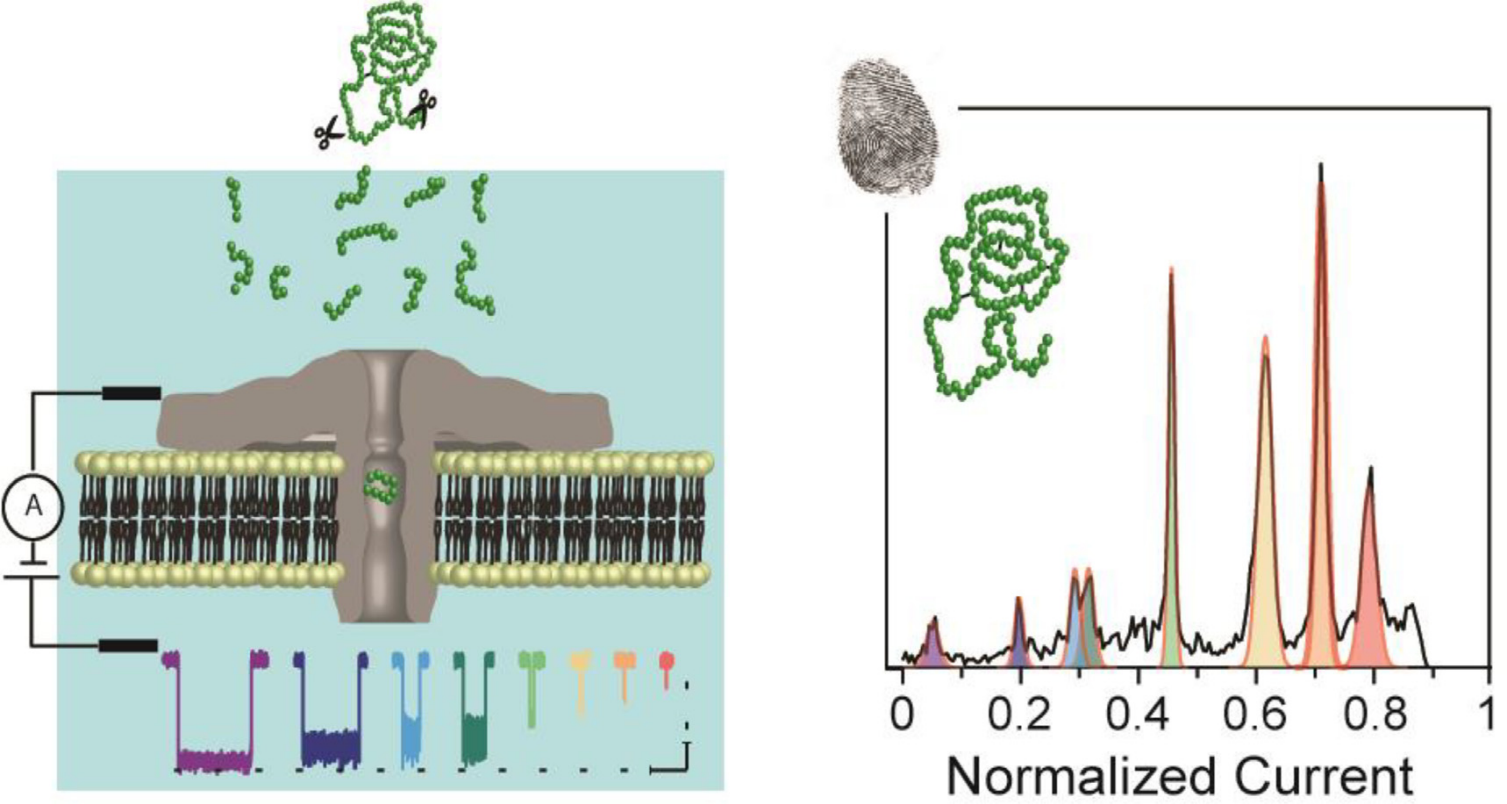
Following protease digestion, peptide fragments of various lengths and sequence can be distinguished as they travel through a nanopore as a function of the normalized current across the membrane, resulting in a unique fingerprint for each protein.

While recent efforts to sequence proteins at the single-molecule level using nanopores have shown promise [28], [34], [35] reading the protein sequence remains a challenge. Considering the complexity of identifying proteins by reading their sequence, a new approach to identify proteins without reading their sequence has recently been demonstrated [36], [37]. This approach consists on identifying proteins by monitoring fragments resulting from the reaction products of a protease through biological nanopores and using protein databases to determine to which protein those fragments correspond, in a manner roughly similar to mass spectrometry (Fig. 3). Specifically, it has been demonstrated that the AeL nanopore enables discrimination between three different proteins with approximately the same molecular mass, opening up the possibility of extending this approach to the identification of proteins irrespective of their size, charge, and structure/conformation [36]. This new approach, which can easily be miniaturized, could replace mass spectrometry as a part of the protein identification workflow. If implemented by a commercial entity, the technology could be made practical and low cost enough for use at point-of-care facilities, as the identification of proteins for health-care applications does not require full sequencing.

#### 2.2.2 Computational design of nanozymes for biomedical applications

**Fig. 4:**
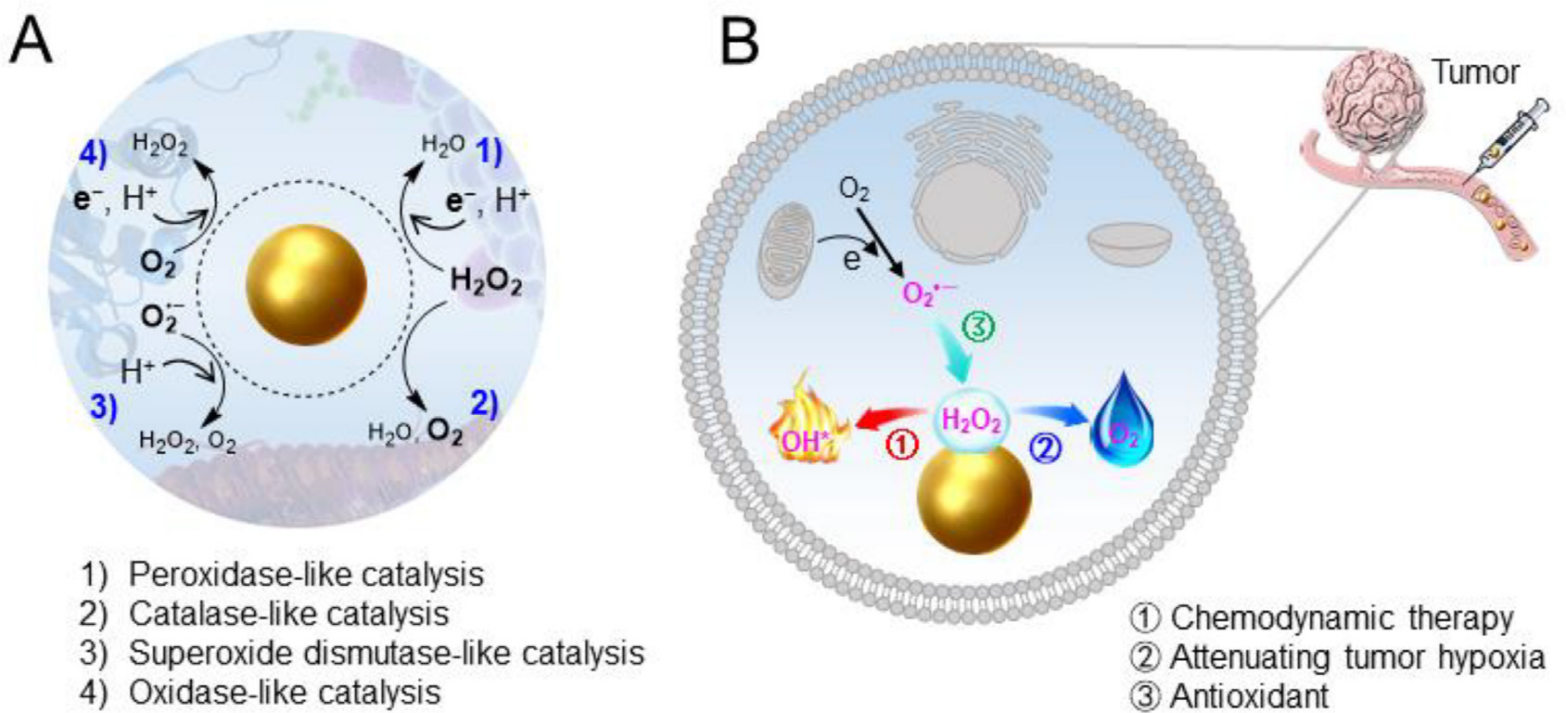
(A) The enzyme-like activities of nanomaterials and (B) potential biomedical applications.

Many inorganic nanomaterials have intrinsic catalytic activities in living cells mimicking those of oxidoreductases, which endow the nanomaterials with intriguing potentials in biomedical applications [38]. These nanomaterials are collectively called nanozymes. For example, the peroxidase-activity of nanomaterials activates H_2_O_2_, which is abundant in tumor cells, to abstract electrons from the surroundings and thus induces apoptosis or necrosis of tumor cells. The catalase-like activity converts H_2_O_2_ into O_2_, which is useful to attenuate tumor hypoxia. The superoxide dismutase-like activity is useful for scavenging O_2_^•−^, providing the nanomaterials with anti-oxidant activity. Owing to their enzyme-like activities, the nanomaterials have potentials in tumor chemodynamic, anti-hypoxia, and antioxidant protection therapies (Fig. 4).

Because specific biomedical applications require that the nanomaterials have high catalytic activities, it is desirable to understand the atomistic-level mechanisms for the catalysis and develop theoretical methods to evaluate the activities prior to experimental applications. Density functional theory calculations have played an indispensable role in studying atomistic mechanisms and structure–activity relationships for catalysts. Using these calculations, the Gao group has studied the mechanisms for the above oxidoreductase-like activities and further developed theoretical methods to predict the peroxidase-, catalase-, and superoxide dismutase-like activities of nanomaterials, paving a road for the application-targeted design of nanozymes [39], [40], [41].

However, challenges still remain. Among them, a big challenge is to improve the substrate selectivity for the nanomaterial catalysis by *de novo* design, and another question is to improve the accuracy of experimental fabrication and characterization of nanomaterials to comply with the design. Other questions like the long-term toxicity of nanomaterials merit further investigation.

### 2.3 Ribosomal frameshifting elements

#### 2.3.1 Programmed ribosomal frameshifting: A potential broad range target for antiviral therapeutics

Human population growth, climate change, and globalization are accelerating the emergence of novel pathogenic viruses. Many viruses use a programmed −1 ribosomal frameshift (−1 PRF) mechanism to direct synthesis of their replicase proteins, a critical switch in their replication program that can be therapeutically targeted [42]. Nearly half a century of research into −1 PRF have provided insight into its biological importance, the molecular mechanisms that drive it, and approaches that can be used to manipulate it towards therapeutic outcomes, with particular emphasis on SARS-CoV-2.

The −1 PRF is a kinetically driven process. Elongating ribosomes are forced to pause over heptameric “slippery sequences” by metastable frameshift stimulating elements (FSEs) on mRNAs, typically pseudoknots [43]. Emerging evidence suggests that the frequency of −1 PRF events is dictated by the extent of structural heterogeneity, or Shannon Entropy, of these FSEs [44]. The idea is that the greater the number of structures that a sequence can sample, the greater the chance that one will be sampled that favors ribosome slippage. The ribosome is also an active player in the frameshift process. The dynamic conformational changes that it undergoes as it transits through the elongation cycle, along with the hydrolysis of GTP by the elongation factors provides the energetic inputs required to overcome the otherwise energetically unfavorable slippage events [43]. While −1 PRF is ultimately a stochastic process, each individual −1 PRF signal is evolutionarily fine-tuned to shift ribosomes at a specific frequency.

First demonstrated using the yeast “Killer” virus system [45], the idea that altering −1 PRF rates negatively impacts viral replication has been broadly demonstrated in retroviruses, coronaviruses, flaviviruses, alphaviruses, and others [42]. Decreases in −1 PRF can result in large, *e. g.,* 3–4 orders of magnitude, decreases in viral copy numbers [46], [47], [48]. The triple-stem loop FSEs of coronaviruses present particularly promising therapeutic targets, as they harbor ring and hole structures conveniently sized to accommodate drug-sized small molecules [49], [50], [51]. Recently, high throughput screens have identified FDA approved drugs that alter SARS-CoV-2–1 PRF efficiency, as well as −1 PRF promoted by other coronaviruses that may potentially emerge into the human population [52], [53], [54]. However, these small molecules were originally optimized to interact with proteins; efforts should be devoted toward developing RNA-specific interactors.

Although numerous academic and private entities are currently targeting the −1 PRF signal of SARS-CoV-2, the challenge of drugging a highly dynamic RNA structural element situates these efforts at the cutting edge of technology. Further, while −1 PRF is an essential regulatory element for many viruses, each viral −1 PRF signal is unique, likely necessitating developing −1 PRF signal-specific drugs for every virus. Additionally, as it is becoming clear that −1 PRF is used to regulate human gene expression [55], [56], the activities of such antiviral agents on −1 PRF in cellular genes will have to be investigated, although, as noted above, the unique nature of each −1 PRF signal may allay this concern. On a positive note, since the −1 PRF signals of each virus are highly conserved, this mitigates worries about drug-resistant bypass mutations. As we try to peer over the horizon to prevent the next pandemic(s), −1 PRF represents an important target to add to the arsenal of antiviral therapeutic strategies.

#### 2.3.2 Unraveling structures and mechanisms of the SARS-CoV-2 RNA frameshifting element by graph theory and molecular modeling

Despite the ongoing vaccination campaigns across the world, we are entering the third year of the COVID-19 pandemic with new waves of infection by SARS-CoV-2 and witnessing mutations that continue to evade our immune system while producing alarming symptoms in increasing cohorts and parts of the world. It has become clear that simple solutions are difficult and that more fundamental scientific knowledge, as well as clinical evidence, is required to address current and future viral infections that inevitably will arise.

Fortunately, the level of scientific cooperation and advances we have witnessed since early 2020 offers hope. Besides successful vaccine development efforts, progress on unraveling the complex and multifarious biophysical aspects of the virus life cycle and infection trajectory has helped us describe how the virus hijacks our own protein-synthesis machinery into making viral proteins efficiently and propose new lines of defense against the deadly disease it carries. These insights into the life cycle of the virus and mode of action are invaluable for further development of drugs and other strategies to combat current and future viral epidemics.

Investigations of the RNA viral agent itself are crucial for understanding how the RNA invader replicates itself, is translated by the human ribosomal machinery, assembles, and synthesizes a suite of viral proteins that enable the continuation of its invasive trajectory. Importantly, RNA-targeting therapeutics and vaccines can disarm the origin of the infection rather than its products and potentially be more effective in the long term. However, the complexity of the RNA molecule and the lagging science about its modeling, imaging, and drug screening, as compared to proteins, poses several challenges. With technological improvements in RNA delivery systems, the rise of CRISPR-based gene editing systems [57], [58], and improved RNA modeling techniques [59], [60], this RNA focus is not only warranted but clearly successful, as evidenced by recent mRNA-based vaccines.

**Fig. 5:**
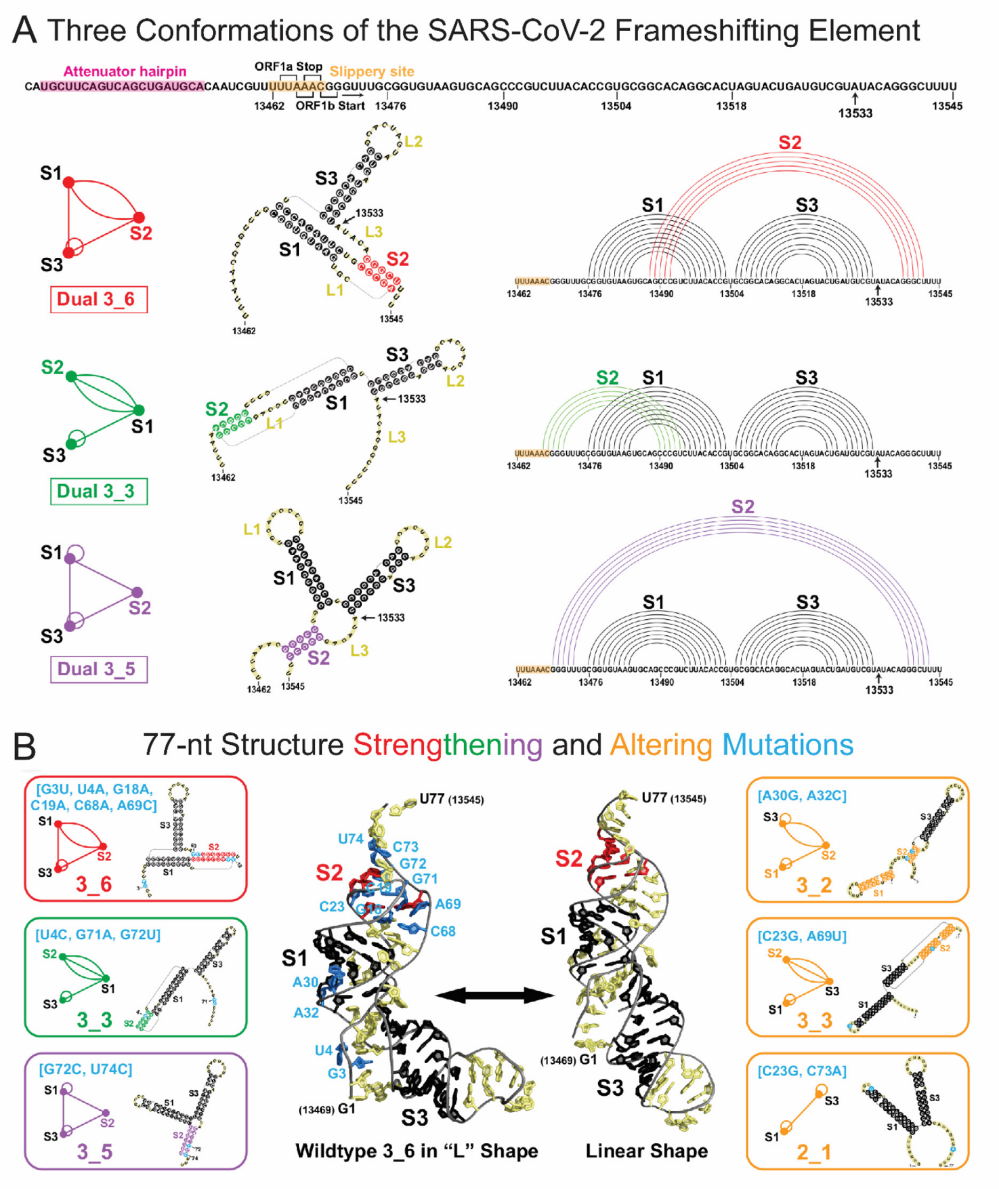
FSE sequence, three relevant 2D structures for the SARS-CoV-2 84 nt FSE (residues 13462–13545) emerging from the Schlick group’s work [84], [96] and mutants that transform the 3_6 motif and stabilize all conformers, using graph based models, 2D structure prediction, SHAPE structural probing, thermodynamic ensemble modeling, and molecular dynamics simulations [97]. (A) The −1 frameshifting moves the transcript UUU-UU*A(Leu)-AAC(Asn)-GGG at the second codon (asterisk) to start as AAA-CGG(Arg), so that translation resumes at CGG. Top: FSE sequence, with the attenuator hairpin region, the 7 nt slippery site, and A13533 labeled (C in SARS). The ORF1a end and ORF1b start codons for the overlapping regions are marked. For each 2D structure, 3_6 pseudoknot, 3_3 pseudoknot, and three-way junction 3_5 (unknotted RNA), we show corresponding dual graphs, 2D structures, and arc plots, with color-coded stems and loops. (B), Left: Mutants that stabilize each conformer; (B), Right: Double mutants that transform the 3_6 form into other RNAs. B, Middle: Straight to L-shape transition captured in the simulation for wild type 3_6 system, which is suppressed in the 3_6 stabilizing mutant.

Of particular interest by many groups is the RNA frameshifting element (FSE), a small region in the open reading frame ORF1a,b region (Fig. 5A) of the viral genome that codes for the polyproteins that initiate the cascade of viral protein synthesis. The FSE is responsible for the crucial −1 PRF mechanism utilized by many viruses, including HIV-1, to handle protein synthesis from overlapping reading frames [61], [62], [63]. A pseudoknot (intertwined hydrogen-bonding), or a stem-loop motif, is believed to be crucial for the requisite pausing associated with frameshifting [63], [64], [65].

When encountering ORF1b, out of register with respect to ORF1a, the ribosome backs up one nucleotide in the 5′ direction to define a different sequence of codons (Fig. 5). Because studies indicate correlations between the FSE conformational plasticity and frameshifting efficiency, it is likely that more complex conformational mechanisms are involved rather than a simple “barrier” [42], [66], [67], [68]. Indeed, the discovery of many alternative conformations of the SARS-CoV-2 FSE [50], [69], [70], [71], [72], [73], [74], [75], [76], [77], [78] suggest a complex conformational and thermodynamic landscape.

The FSE’s crucial role in viral protein synthesis makes it an excellent target for therapeutic intervention [42], [79], [80]. Already, small-molecule agents such as 1,4-diazepane derivative 10 (MTDB) (originally designed for SARS-CoV [68], [81], [82]), fluoroquinolone antibacterial merafloxacin [74], and a phenyl thiourea C5[83] were found to hamper SARS-CoV-2 frameshifting. However, a mechanistic understanding of the process and the drug effect is unknown. Complexity is inherent in the makeup and variability of the FSE region. The 84-residue SARS-CoV-2 FSE (13462–13545 of the 29,891 nt RNA genome) contains a 7-residue slippery site (UUUAAAC) and a 77-residue conformationally flexible region (Fig. 5). Besides the folds and mechanisms of the RNA FSE itself, important interactions are involved between the FSE and the ribosome during viral protein synthesis.

The Schlick group focuses on better understanding the conformational landscape of the FSE using a combination of complementary graph-based modeling and chemical reactivity and other experiments*.* Already, several groups have explored FSE structure by modeling [49], [69], [77], [78], [84], in vivo Selective 2′-Hydroxyl Acylation by Primer Extension (SHAPE) [85], [86] and DMS structure probing experiments [50], [70], [71], [72], [73], [74], [75], [76], NMR [87], Cryo-EM [50], [51], and other biophysical mutational profiling and scanning approaches [82], [88], [89].

Many have characterized the FSE of SARS-CoV-2 as a 3-stem hairpin (H-type) pseudoknot with colinear Stems 1 and 2 intertwined via a pseudoknot and perpendicular Stem 3. This association has persisted from the SARS-CoV FSE characterization [82], which differs in only one base from the SARS-CoV-2 FSE (residue A13533 in Covid-19 is C in SARS, Fig. 5). However, depending on the modeling software and experimental technique, alternative secondary structures have been reported for SARS-CoV-2, both pseudoknotted and unknotted [50], [69], [70], [71], [72], [73], [74], [75], [76], [77], [78].

Developing further and applying the RNA-As-Graphs (RAG) graph-theory tools for handling pseudoknots [90], [91], [92], [93], [94] to the SARS-CoV-2 FSE, the Schlick group has designed double mutants, shown in Fig. 5B, that dramatically alter the FSE conformation (see [84] and accompanying New & Notable commentary [95]). These mutations define target residues for drug binding and gene editing. By further probing the FSE structure as a function of length and performing chemical reactivity experiments with SHAPE, we have described alternative forms of the FSE [96]. Notably, the combined complex conformational landscape has *three viable structures* of the FSE: two 3-stem pseudoknotted RNAs (3_6 and 3_3 in the dual graph notation), and one unknotted, 3-way junction RNA (3_5) (Fig. 5B). The flexible Stem 2 may be involved in a switch between these conformations and associations with the ribosome during protein translation, as well as define a co-transcriptional kinetic folding trap. For whole genome constructs, a stem-loop motif (2_2 graph) may also compete with these forms. In addition, we confirmed experimentally that other designed FSE mutants stabilize one conformation over all others [97], lending confidence in these mutational transformations. These conformation-specific mutations may be particularly effective when used in combination with anti-viral compounds that target a specific FSE form. The Schlick group’s recent molecular dynamics studies of these alternative conformations at various lengths suggest key transition states that are shared by all three FSE conformations and, importantly, pathways for inter-conversion among them [97]. These studies also point to several therapeutic avenues, including interference with FSE transformations, FSE/ribosome interactions, and FSE folding. Ongoing research is addressing these possible therapeutic avenues as well as the dynamic mechanisms associated with frameshifting.

### 2.4 RNA structure predicton and assessment

#### 2.4.1 End-to-end prediction of RNA base pairing structures through deep transfer learning of evolution and co-evolution information

Predicting RNA secondary structure has long been dominated by folding-based algorithms, which require a scoring function coupled with a minimum-searching algorithm. These predictions, however, are often limited to nested and stacked canonical base pairs (i.e., AU and GC Watson-Crick and GU wobble base pairs), while ignoring other hydrogen-bonded bases stabilized by tertiary interactions. The ignorance reflects the difficulty to predict “tertiary” base pairs without knowing three-dimensional structures. Structure-stabilized base pairs include un-nested (pseudoknot), noncanonical and isolated lone base pairs as well as base triplets. Thus, a gold-standard dataset for these “tertiary” base pairs requires experimentally determined three-dimensional structures. However, there are only a few thousand RNA structures that have been solved so far. Such a small number of RNA structures makes it challenging to apply deep learning to RNA structure prediction.

Recently, Singh et al. showed that the above problem can be addressed by using a highly accurate but approximate database of RNA secondary structures for initial training [98]. The initial training is followed by transfer learning with a small dataset of full base-pairing structures derived from RNA structures. The resulting method called SPOT-RNA showed that a single sequence (i.e.*,* without information of homologous sequences) can provide a significant improvement over the stagnant accuracy of secondary structure prediction by folding-based algorithms in recent years. The most significant improvement is in “tertiary” base pairs such as pseudoknots, noncanonical and lone base pairs.

**Fig. 6:**
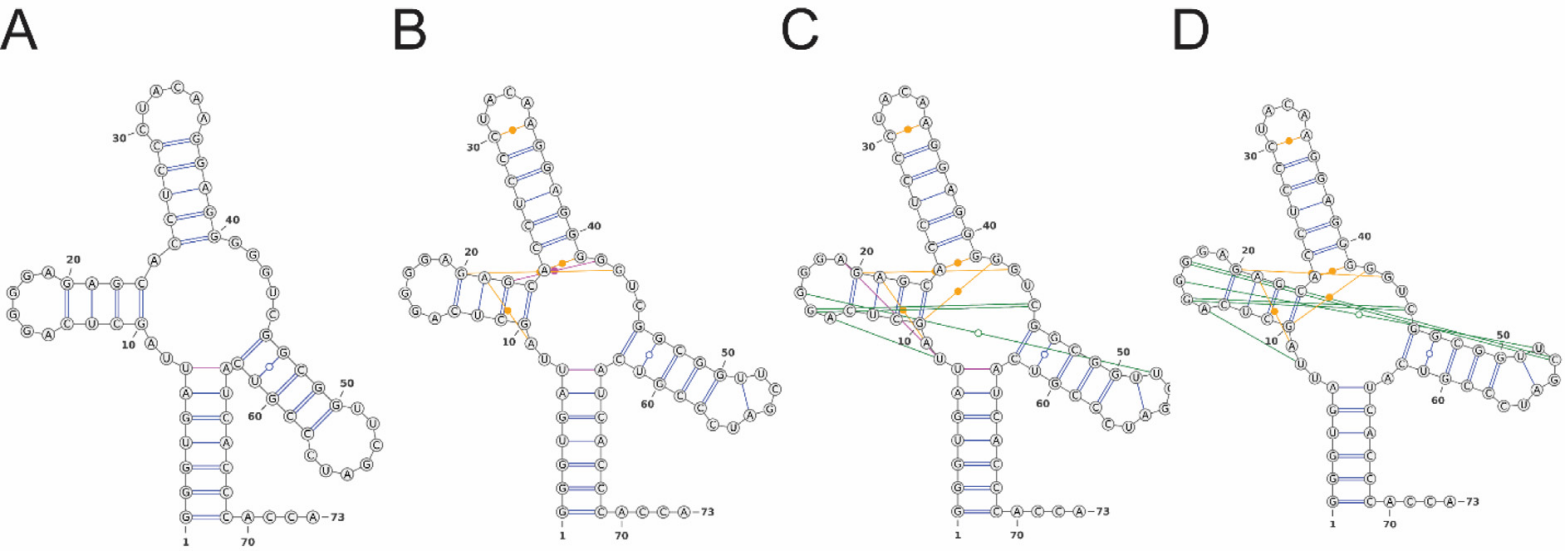
Base pair prediction for 70S ribosome (PDB 6CAE, chain 1Y) by (A) CentroidAlifold [PR = 95, SN = 67], (B) SPOT-RNA [PR = 93, SN = 83], and (C) SPOT-RNA2 [PR = 94, SN = 97], compared to (D) the native as labelled. All false positives are shown in red. Tertiary base pairs (noncanonical base pairs in orange and pseudoknots in green) are the most visible improvement from the traditional folding algorithm (CentroidAlifold, limited to canonical base pairs in blue) to SPOT-RNA without evolution information and SPOT-RNA2 with evolution information. Copyright (2021) Oxford University Press.

To further improve the above prediction, it is necessary to incorporate sequence conservation and co-variation information as in proteins. However, unlike proteins, 4-letter coded RNA can easily lose their sequence identity in evolution. Thus, it is necessary to incorporate structural information in homology search as has been done in INFERNAL [99], which searches homology sequences using a secondary-structure-based covariance model. RNAcmap is the first fully automatic pipeline that performs homology search, multiple sequence alignment, and direct coupling/co-mutational analysis in a single run [100]. Incorporating sequence profiles and mutational coupling generated by RNAcmap yields another significant improvement in RNA secondary structure prediction (SPOT-RNA2 [101]) and distance-based contact prediction (SPOT-RNA-2D [102]) for those RNAs with a significant number of homologous sequences, in particular. One example was shown in Fig. 6, where SPOT-RNA2 provided accurate prediction of tertiary base pairs. The result highlights the usefulness of deep learning and multiple sequence alignment for the problems related to RNA structure prediction. RNAcmap, SPOT-RNA, SPOT-RNA2 and other RNA/protein-related tools (SPOT-RNA-1D for backbone structure [103] and RNAsnap2 for solvent accessibility [104]) are available for download or as online servers at https://sparks-lab.org.

Despite the above progress, many challenges remain. For example, RNA homologies remain difficult to find, for long RNA sequences (>1000 nucleotides) in particular [100]. Moreover, even with perfect contact maps as restraints, many RNA structures remain elusive [103] because RNA backbone structures have a much higher degree of freedom (6 torsion angles) than protein backbone structures (2 torsion angles). Better energy functions are certainly needed [105]. More importantly, the number of 3D structures in protein databank is insufficient for typical deep learning techniques. Thus, it is likely that a combination of deep learning with RNA-specific scoring/energy function is required to make significant progress in the foreseeable future [106].

#### 2.4.2 Cryo-EM reveals conformational dynamics in a multifunctional viral RNA

Development of new nanotechnologies based on engineered RNA structures depends on knowledge of the fundamental properties of RNA folding and its conformational dynamics [107]. The modular architecture of RNA can be exploited to rationally build RNA structures [108], [109], but we need to solve the structures of more, and more dynamic, RNA structures to understand underlying principles and discover conformationally dynamic ‘building blocks’ for use in RNA nanotechnology.

Viruses ubiquitously use structured RNA elements as part of their overall infection strategy, and for regulating the formation of the capsid [110], [111], [112], [113]. Many structured viral RNA elements are multifunctional, a feature that may depend on conformational changes programmed into the RNA structure. Thus, explorations of these RNA elements can yield new tools for RNA nanotechnology while revealing fundamental features of RNA folding and dynamics. Although traditional structural methods are ill-suited for large, structurally dynamic RNAs, advances in cryo-electron microscopy (cryo-EM) may provide a new way to explore these RNAs.

Examples of structured, multifunctional, and perhaps conformationally dynamic RNA elements are the tyrosine-accepting tRNA-like structures (TLS^Tyr^). TLS^Tyr^ are found at the 3′ end of specific single-stranded positive-sense RNA viral genomes [114], [115]. They are aminoacylated by the host cell’s tyrosine aminoacyl tRNA synthetase (Tyr-AARS) and regulate several viral processes. These characteristics suggest TLS^Tyr^ are tRNA mimics that undergo programmed conformational changes, but their structure had remained elusive for decades.

**Fig. 7:**
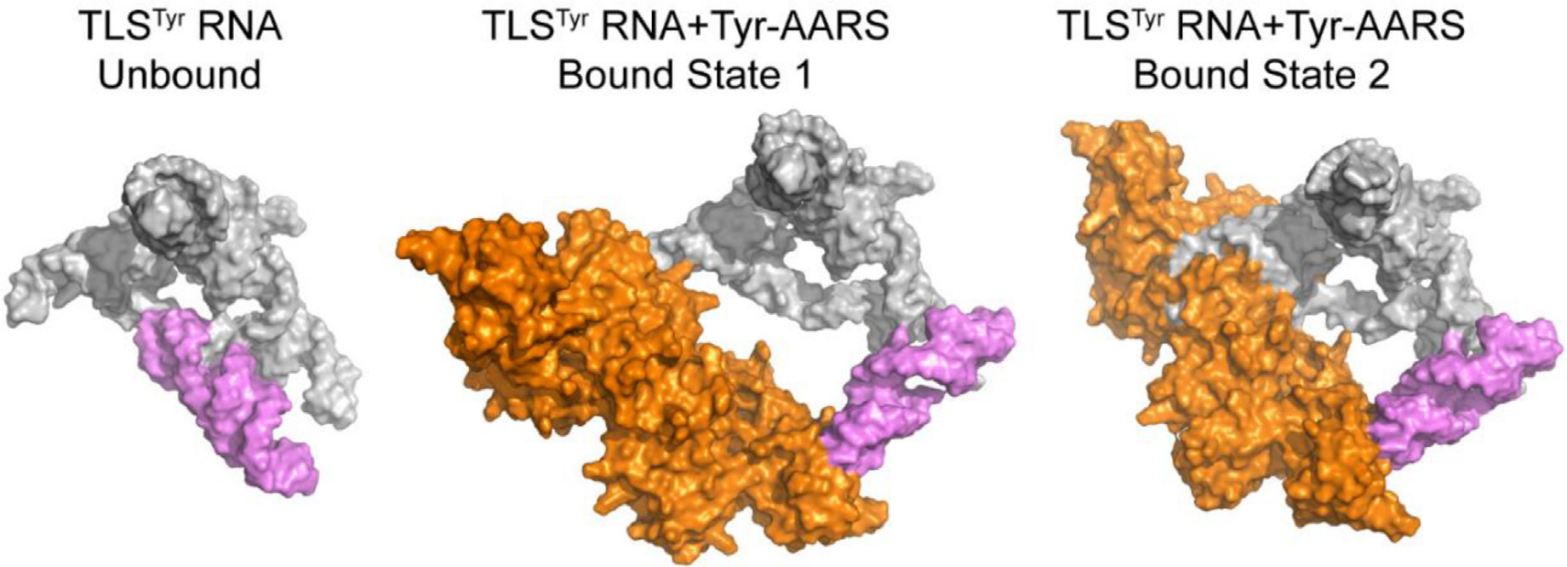
Structure of a TLS^Tyr^ RNA (grey and purple) in the unbound form (left) and bound to a Tyr-AARS, which is colored orange (center and right). Solvent-accessible surfaces are shown. The purple TLS^Tyr^ RNA domain is dynamic relative to the rest of the molecule (grey), adopting different positions in the unbound versus bound states.

The Kieft group has used single-particle cryo-EM to solve the structure of a representative TLS^Tyr^ RNA (∼55 kDa) in both its unbound form and bound to Tyr-AARS (Fig. 7) [116]. The unbound RNA comprises a complex structure of multiple helical elements emanating from a central junction. Surprisingly, the structure does not directly mimic tRNA, suggesting it must change conformation to productively bind the Tyr-AARS. Further analyses revealed that the part of the RNA that acts as the anticodon loop mimic is within a conformationally mobile domain. The structure of the TLS^Tyr^ RNA in the Tyr-AARS-bound state subsequently showed that this mobile domain adopts a position at a roughly right angle to its position in the unbound state. This mode of binding differs dramatically and unexpectedly from that of an authentic tRNA.

These findings show that programmed conformational dynamics are an inherent and necessary component of TLS^Tyr^ RNA’s ability to hijack the host cell synthetase. Furthermore, there exist two distinct bound states, one in which the TLS^Tyr^ RNA is making limited contact with the enzyme and one in which the 3′ end of the RNA is ‘pulled into’ the enzyme’s active site. Although we cannot ascribe an order of events to these states, they suggest a multistep mechanism of binding that may be more generally applicable. Overall, these studies show the power of cryo-EM to detect and characterize conformational changes in RNAs that have been largely intractable by other methods.

#### 2.4.3 Correlating SHAPE data to RNA 3D structure

The talk from Shi-Jie Chen’s research group from the University of Missouri, Columbia, focused on a novel method for data-assisted RNA structure prediction. As a readily available alternative to X-ray footprinting of RNA for assessing secondary and tertiary structures which does not require the use of a synchrotron, selective 2′-hydroxyl acylation analyzed by primer extension (SHAPE) provides insights into the flexibility of nucleotides [117], [118]. A SHAPE ligand such as 1-methyl-7-nitroisatoic anhydride (1 M7) reacts with nucleotides in the flexible regions of RNA (flexible loops) and does not react with nucleotides in rigid regions (helices, rigid loops). SHAPE is known to supply effective constraints for the prediction of a 2D structure (base pairs) [85]. However, nucleotide flexibility is determined by atomic interactions, which is 3D. Thus, SHAPE reactivity is intrinsically tied to the 3D structure of RNA. The Chen lab has developed, SHAPER [119], [120], [121], a web server (URL: https://rna.physics.missouri.edu/shaper/) that can compute the SHAPE profile from a 3D RNA structure. Combined with computer simulations that can generate a pool of candidate 3D structures, SHAPER would provide a novel, SHAPE data-driven, method to efficiently identify the native structure or near-native structures by removing SHAPE-incompatible structures from the pool of candidate structures.

The key product of the model is a new estimator of SHAPE reactivity for each nucleotide in a given 3D structure [85], [119]. The main driver of the prediction is the energy-like score that connects the SHAPE reactivity profile to the energetics of the nucleotide. This SHAPE energy score is related the 3D interaction energy that combines the effects of base pairing and stacking to estimate the interaction strength for a nucleotide. This score also accounts for several key factors including ligand accessibility, sugar conformation, and the effect of terminal residues due to alterations of the terminal regions of the RNA during the experiment. Because a SHAPE reaction occurs at the 2′-OH, the SHAPE ligand has to access the space between the nucleotide being scored and the nucleotide directly downstream, so the interaction strength of these two nucleotides with other members of the RNA and the 5′ to 3′ polarity of the nucleotide interactions are very important to the ability of the SHAPE ligand to react. Furthermore, Chen lab found that including the non-nearest-neighbor interactions of these nucleotides is also important in estimating the SHAPE reactivity.

**Fig. 8:**
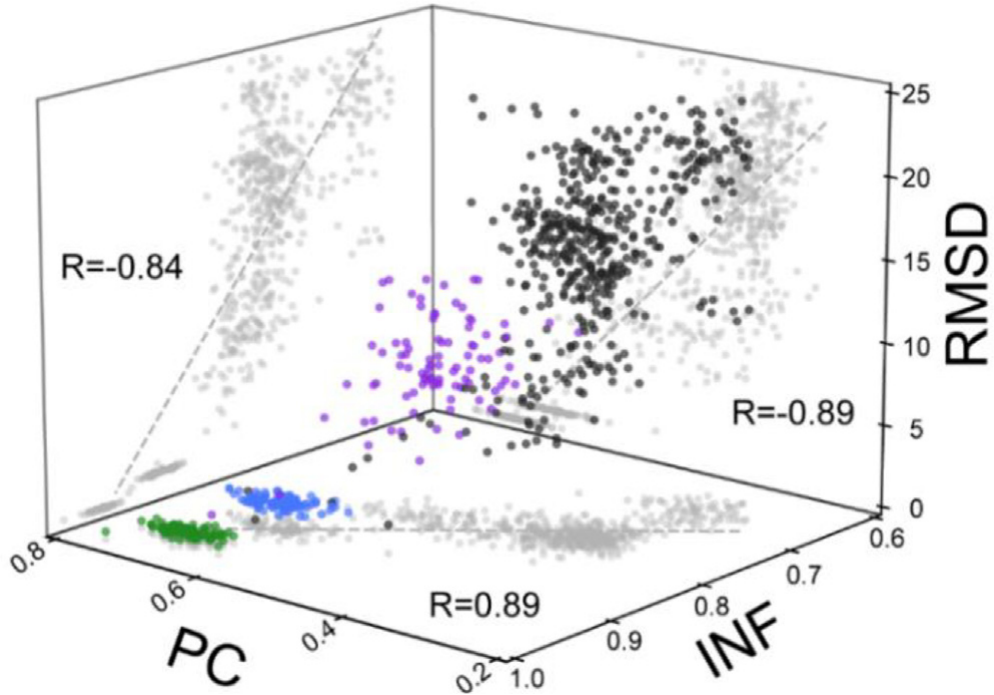
The figure shows the trends between the Pearson correlation, INF and RMSD for Lysine riboswitch (PDBID: 3DIG). The INF is a measure that captures native interactions, so the reference native structure has an INF of 1. The RMSD is the root mean squared deviation of the structure, so the reference native structure has an RMSD of 0. Structures in the green region are the decoys generated from a simulation of the native RNA with the backbone and base pairs restrained. The blue region represents a similar ensemble of structures, except that the base pairs are no longer restrained while the backbone atoms are restrained to maintain the global folding. Structures in the purple region are the decoys generated from coarse-grained simulations with the base pairs enforced, but no backbone restraints. The black region shows an ensemble of alternative (nonnative) low-energy 2D structure decoys generated from coarse-grained simulations. The projections of the decoy structures are shown as shadows in each 2D plane. We see that as the INF increases and RMSD decreases, i.e., as the structure approaches the native state, the Pearson correlation between the SHAPER prediction of the SHAPE reactivity and the experimental SHAPE reactivity of the structures increases, indicating that the model is successful in estimating the SHAPE reactivity for decoy structures. This may be used for predictive purposes. By sieving low-energy decoys, the model can improve predictions RNA structure prediction by removing structures that are incompatible with SHAPE data.

Overall, this new model displays substantial promise in the assessment tested. For a structure, the Pearson correlation is calculated between the predicted SHAPE profile and the experimental profile. The result (Fig. 8) of the Spearman rank correlation between the Pearson correlation and the interaction network fidelity (INF) show that the model is able to discern between near-native and non-native structures with a high degree of accuracy. Taken together, these results suggest that the model is both sensitive and accurate in identifying the native or near-native 3D folds of an RNA. Combined with SHAPE data, the model would provide a powerful tool for RNA nanoparticle design based on the desired RNA native structures [122], [123], [124], [125], [126], [127], [128], RNA conformational changes [129], [130], [131], [132], and RNA-ligand interactions [133], [134], [135].

There are two major challenges to the SHAPER model. First, the SHAPE data used to train the model are from various systems and may rely on different data processing of the raw data. Therefore, the model may suffer from the quality of the training data set. This has been shown by a previous attempt of using machine learning to predict the SHAPE profile from a 3D RNA structure [136]. Second, the correlation between intra-molecular interactions and SHAPE reactivity is not fully clear, which would directly impact the reliability of the model. In future developments, more high-quality SHAPE data needs to be collected for different RNA structures, in particular, for RNA nanoparticle structures. With such data, the SHAPR model will be refined to more effectively and accurately sieve incorrect 3D folds and identify the correct native structures for RNA nanoparticles. Furthermore, future development of the SHAPER model should rely on a rigorous model for intramolecular interactions, in particular, for the non-canonical base-pairing interactions in different tertiary structural motifs. Better models of the interactions demand more biophysical data on RNA structure and stability.

#### 2.4.4 Integrating molecular dynamics simulations and SAXS data

Small angle X-ray scattering (SAXS) experiments are valuable in providing information about size and shape of RNA molecules and thus optimally complement other biophysical techniques [137], [138]. Given their low resolution, SAXS data must be integrated with accurate modeling tools so as to provide detailed structural information. For a single molecule in vacuum, frozen in a single conformation, scattering intensities are straightforwardly related to the radial Fourier transform of the atomic coordinates. However, for biomolecules in solution, two additional factors might make the interpretation more difficult, namely (a) the contribution of the solvent and (b) the presence of multiple alternative conformations in the same buffer (RNA dynamics).

Molecular dynamics simulations allow for RNA dynamics to be investigated at atomistic resolution and are currently a standard tool for structural biology studies [139]. Notably, they can be combined with SAXS data so as to provide an interpretation of the experiment at atomic resolution [140]. In theory, they allow both factors mentioned above to be considered. Solvent effects can indeed be included by explicitly subtracting a separate buffer simulation, similarly to what is done in experiments [141], [142]. At the same time, the presence of multiple conformations naturally arises from the spontaneous structural fluctuations, which can be directly averaged. Given the typical timescales required for RNA conformation changes, this can only be obtained by using suitably designed enhanced sampling methods [143]. These two ideas have been however rarely combined.

**Fig. 9:**
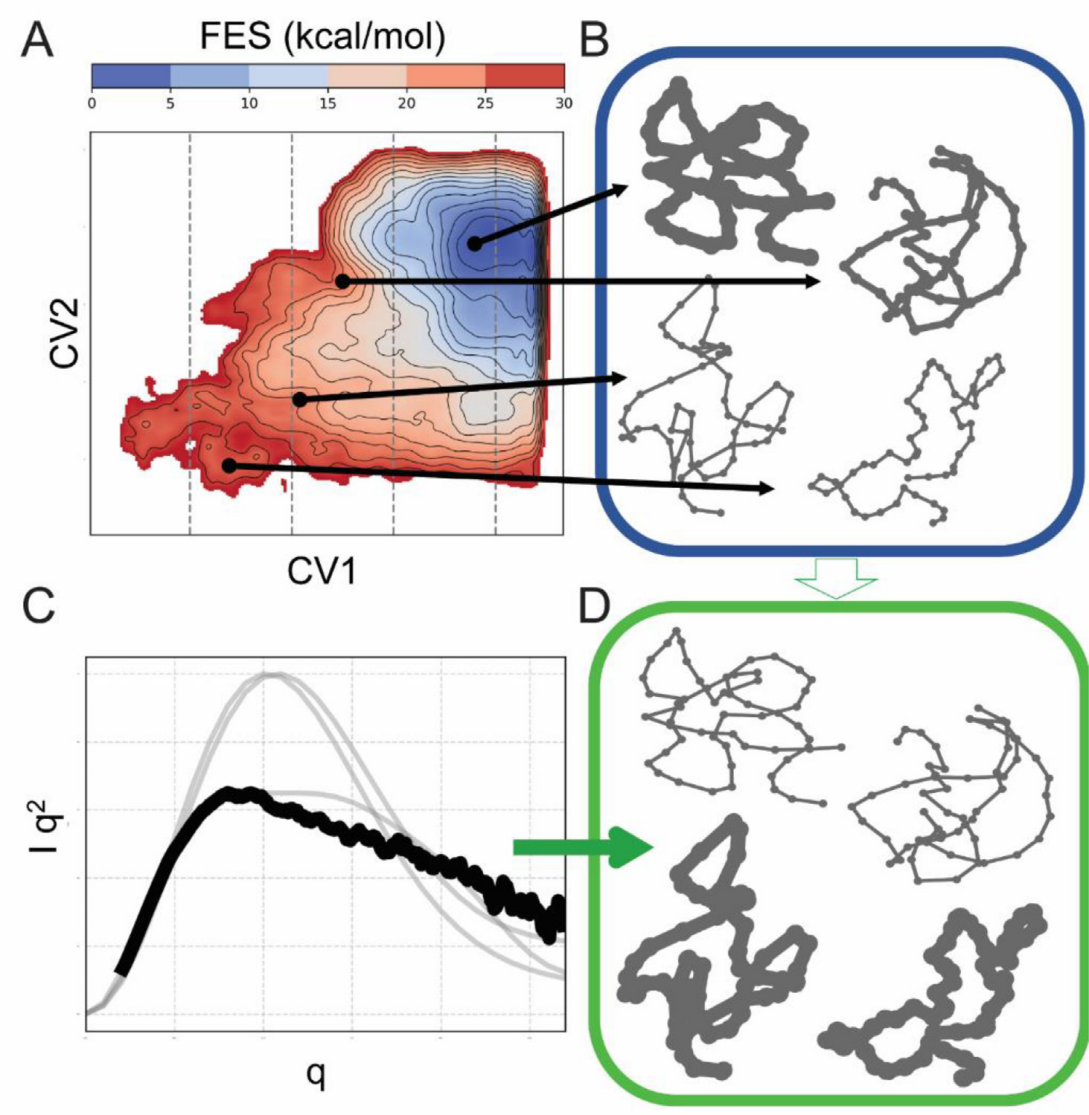
Integrating enhanced sampling simulations and SAXS data. (A) Molecular dynamics simulations with enhanced sampling techniques allow the extraction of a free energy surface (FES) as a function of one or more collective variables (CV1 and CV2). (B) The generated ensemble contains both compact and extended structures, and associates them with a population. (C) SAXS experiments at different ion concentrations provide a spectrum that is affected by solvent effects and ensemble averages. (D) The combination of molecular dynamics and experiments allow RNA dynamics to be reconstructed at atomistic resolution. Copyright (2021) Oxford University Press.

In a recent work [144] (see also Fig. 9), the Bussi group has shown how solvent effects and RNA dynamics can be brought together to characterize the ion-dependent population of compact and extended states of the GTPase-associated center, an RNA molecule involved in protein translation for which SAXS data were previously reported [145]. Specifically, an enhanced sampling method [146] was used that encourages the exploration of structures with heterogeneous SAXS spectra, by quickly estimating spectra on-the-fly without including solvent effects [147]. This allowed to generate an ensemble consisting of a mixture of compact and extended structures. The SAXS spectra for the generated ensembles were then reconstructed by explicitly including water contributions and modeling the experimental buffer subtraction [141], and use the maximum entropy principle to reweight the results so as to match experiments [148]. The results show that different populations of compact and extended states are required based on the type and concentration of ions present in the buffer.

Overall, these results show that both conformational dynamics and solvent effects play an important role in SAXS spectra of RNA, and indeed, neither of the two effects can separately account for the experimental results. The importance of the solvent might be ascribed to the highly charged nature of RNA. At the same time, the observation that rich and heterogeneous conformational ensembles are required to match experiments underlines the importance of RNA dynamics in analyzing data obtained in solution.

Given the relative simplicity of small (and wide) angle X-ray scattering experiments (SAXS and WAXS) when compared to other biophysical techniques, as well as their capability to capture dynamical effects, the application of these techniques to RNA systems is gaining momentum. At the same time, several methods are emerging to combine them with molecular modeling techniques so as to extract the maximum amount of information. We believe that this field will see other methodological developments in the next few years. In addition to methods based on maximizing entropy, such as the one discussed above, other techniques based on the maximum parsimony principle, such as sample-and-select, have been recently applied to RNA [149]. These techniques have the advantage of reporting a limited number of structural models, and thus provide more interpretable results, at the price of ignoring a possibly relevant fraction of conformation dynamics. A future challenge in the field is certainly the synergistic integration of these ideas so as to combine their benefits. Finally, it is important to recognize that the applicability of atomistic molecular dynamics simulations to medium-sized (50–100 nucleotides) RNAs is extremely expensive, especially in the most interesting cases where conformational dynamics is important. The field will thus certainly benefit from the development and availability of novel enhanced sampling methods based on the application of machine learning techniques [150]. An alternative approach is that of using coarse-grained models that have an inherent advantage when compared to MD in their capability to quickly generate large sets of heterogeneous structures [151]. Hence, we believe that future research should be aimed at developing methods to increase the accuracy in the back-calculation of SAXS and WAXS spectra when using coarse-grained representations of RNAs.

### 2.5 RNA nanotechnology

#### 2.5.1 The dynamic property of RNA leads the emerging field of RNA nanotechnology

RNA folds into thermostable structures and nanocomplexes with defined stoichiometry, size, and shape from one or multiple RNA oligo strands [152], [153], [154], [155], [156], [157], [158], [159], [160], [161], [162]. RNA ensemble structures hold a higher level of apparent entropy (S), or structural disorder than DNA, despite RNA possessing more a compact helical region [107], [152], [161]. Helical regions of RNA are highly structured and inflexible, but other elements, such as loops and bulges, are capable of dynamic motions capable of interacting with small molecules and receptors via induced fit models [153]. The high level of entropy and the ability for dynamic motions allow for RNA to have the property of movement, motion, and deform shapes much like an amoeba. This amoeba-like property has often been encompassed in a worm/snake-like chain structural model and possesses unique biodistribution properties [163], [164].

Methods for examining the motion and movement of RNA utilize nuclear magnetic resonance (NMR) [154], [155], [156], [157], [158], [159], X-ray crystallography [165], [166], single-molecule imaging microscopy [167], [168], [169], and cryogenic electron microscopy (cryo-EM) [169], [170].

**Fig. 10:**
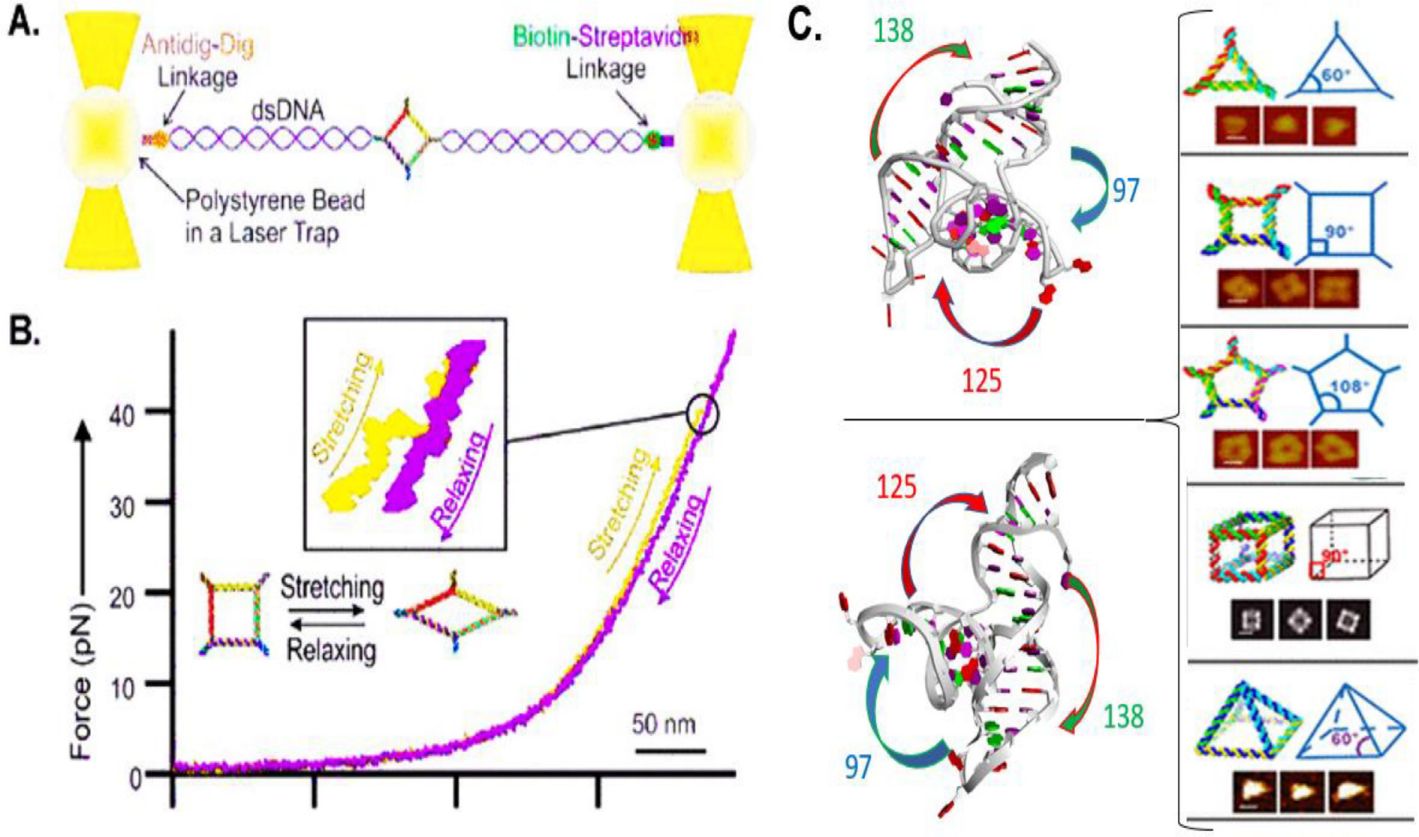
RNA nanoparticles stretched via dual trap optical tweezer by Guo and colleagues. (A) Dual trap optical tweezers with tethered RNA construct between two dsDNA handles via affinity linkers. (B) Control force–extension curve for stretching (red) and relaxing (black) of RNA nanoparticle as a square. Inset: Point at which the nanoparticle undergoes conformational change in response to force. [171] (C) RNA nanostructures constructed through using the dynamic property of pRNA 3WJ its natural angles of 97, 138, and 125 respectively, tunable past its these natural angles to 60°, 90°, and 108° respectively. [172] (A-B) Reprinted (adapted) with permission from Ghimire et al 2019. Copyright 2019 American Chemical Society. (C) Copyright 2014 Oxford University Press.

RNA moves to change conformations in the presence of a ligand during the binding of proteins, or when undergoing co-transcriptional dynamic folding and restructuring, demonstrating movement during enzymatic behaviors in accordance with the induced fit model of substrate ligand interactions [153], [160], [161], [162], [166]. Besides mRNA, rRNA, and tRNA, cells contain many other noncoding RNA for critical functions in the regulation of basic cellular functions [173], [174], [175]. The dynamic nature of RNA results in its motile and deformative behavior which is interacting with a large variety of cellular functions aside from post-transcriptional and translational functions. This deformative property is highlighted in Fig. 10. Conformational transitions change base-pairing, breathing within complemented strands [176], and pseudoknot formation at a 2D level [177], as well as the induced-fit [178] and conformation capture at the 3D level [179] which are important for biological functions including regulation, translation, and catalysis. The dynamic conformational transition follows the nearest neighbour principle to determine an ensemble of transient states with respective Gibbs free energies sitting around the “lowest” Gibbs free energy state which are adopted at any moment [180]. The dynamic, motile, and catalytic activity has led to a belief that RNA is the origin of life in the RNA world hypothesis, underscoring the interest in understanding RNA structural dynamics [181]. This deformative property of RNA nanoparticles enhances their trans vascular permeability past the leaky blood vessels associated with angiogenesis while also rapidly passing the glomerular membrane for rapid body clearance. The biodistribution of RNA nanoparticles can be further improved by the incorporation of ligands for entry into solid tumours with extensive vascular networks [164]. The negative charge of RNA decreases the toxicity of drugs carried by this platform by preventing nonspecific binding to the negative charge cell membrane while enhancing the solubility of hydrophobic drugs for favourable hemodynamics [182]. These unique properties of RNA nanoparticles and the mechanism of RNA dynamic, motile and deformative properties were presented at the meeting to welcome RNA therapeutics as the third milestone in pharmaceutical drug development.

#### 2.5.2 Strand displacement in nucleic acid nanotechnology

Strand displacement is a reaction between DNA or RNA strands that is essential to dynamic nucleic acid nanotechnology, and has been used in nucleic acid circuits designed for molecular computing, as well as in synthetic biology and molecular sensing [183]. Furthermore, there is increasingly more evidence that strand displacement is also involved in numerous naturally occurring processes, such as during RNA cotranscriptional folding or during RNA invasion into double-stranded DNA in the CRISPR-Cas9 complex [184].

**Fig. 11:**
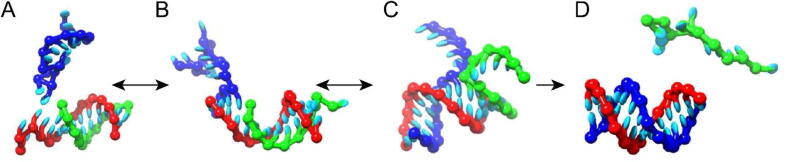
A schematic representation of the toehold-mediated strand displacement reaction that was previously simulated with a coarse-grained RNA model. (A) Invader starts to bind to toehold. (B) Invader strand fully bound to the toehold. (C) Invader starts to replace base pairs between the incumbent and the substrate. (D) Invader fully replaces all bonds between the incumbent and the substrate.

The Sulc group has studied toehold mediated strand displacement reaction (TMSD) [185], [186], [187]. It consists of an invader strand that binds to a complementary strand (substrate) that has been previously bound to another complementary strand (incumbent) (Fig. 11). The substrate strand has a single-stranded overhang (toehold) to which the invader bounds prior to removing the incumbent.

While the kinetics and thermodynamics of the TMSD reaction between DNA strands has been studied in detail for DNA nanotechnology via computational and experimental studies, there has not been a systematic study of such systems experimentally carried out when RNA strands are involved. Experiments have also not been carried out for hybrid reactions, where either RNA invades a DNA duplex, thus creating RNA-DNA hybrid, or when DNA strands invades an RNA duplex. The Sulc group has performed an experimental study where they varied the length of the toehold for RNA TMSD, as well as introduced mismatches between the invader and the substrate. Through this work, it was found that increasing toehold length speeds up the reaction exponentially, as was previously observed for DNA. Introducing mismatches slows the reaction, and the closer the mismatch is to the interface of the toehold / incumbent strand, the larger the slow-down. For a six-nucleotide toehold, it was observed that RNA invading an RNA duplex is faster than RNA invading a DNA duplex. For a toehold of length 3 nucleotides, they found RNA invading DNA to be faster than RNA invading RNA.

It should be noted that these studies are just a first step towards understanding the strand displacement kinetics that involves hybrid DNA-RNA systems. Such systems are still not integrated into available simulations and the design tools that our community uses to design and test strand displacement circuits. The studies which involve invasion (of DNA or RNA) into DNA-RNA hybrid circuits have not been performed yet, and testing of effects of different sequences and positioning on the 5′ or 3′ end of substrate is also needed for better understanding of RNA TMSD reaction. Finally, while the thermodynamics of DNA and RNA duplexes has been carefully studied over the years, and is currently incorporated into widely used dynamic programming tools for secondary structure prediction such as NUPACK or RNAStructure, the free energies of fully complementary DNA-RNA hybrids were measured in the above work. The thermodynamics of mismatches in DNA-RNA hybrids has still not been measured.

#### 2.5.3 Rationally designed nucleic acid nanoparticles (NANPs) with regulated immune recognition

As was revealed by the COVID-19 pandemic, there are numerous biological challenges that we are not prepared for [8], [188]. Nevertheless, while the same risks still exist for many other pathogens and diseases, there is now an enormous potential available for accelerated solutions that can be provided for future health threats.

With traditional small molecule drugs, there are two main components -- the dianophore that defines drug’s biodistribution and pharmacokinetics, and the pharmacophore that determines its targeted function and biological responses. Therefore, even minor changes to a chemical composition of a small molecule drug can have drastic effects on its function and distribution and consequently, would require its complete re-evaluation [189]. In contrast, the separation of the dianophore and pharmacophore components yields a more modular therapeutic approach. In the case of therapeutic nucleic acids (TNAs), the backbone chemistry and the sequence define the dianophore and the pharmacophore components, respectively. This modularity allows for a tighter control over therapeutic characteristics of the drug and by changing ether chemical makeup of the backbone or the sequence of TNAs, the new therapeutic effect can be predicted and tuned as required for specific functions.

On top of being used as therapeutics, nucleic acids can now be employed as nanoscaffolds to coordinate simultaneous delivery of multiple different TNAs that are intended for synchronized action in the same diseased cell [190], [191]. These nucleic acid nanoparticles, or NANPs, are rationally designed to self-assemble into specific structures [192] that gain pre-defined architectural and physicochemical parameters [193]. Furthermore, NANPs can be programmed to carry multiple functionalities embedded in their structures that all together can be activated to target multiple pathways in a particular disease.

**Fig. 12:**
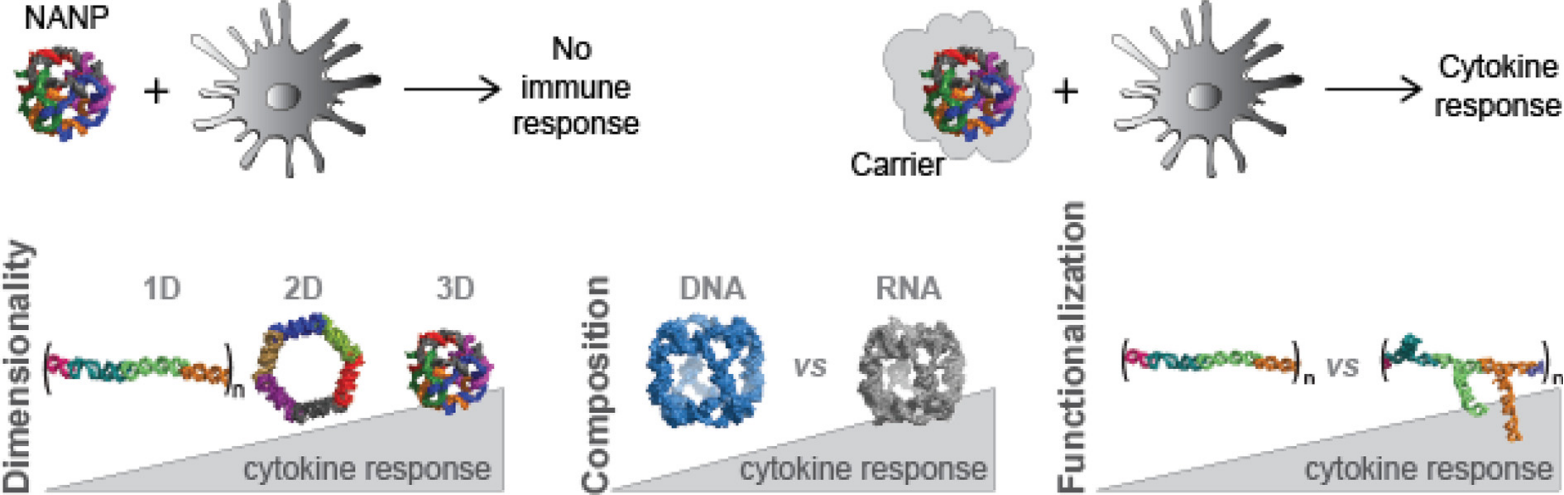
The immunorecognition of NANPs is defined by their structure, composition, functionalization and require the use of delivery agents.

Due to the intended applications, it becomes important to understand how NANPs interact with the human innate immune system, evolutionary equipped with the diverse set of pattern recognition receptors (PRRs) that act specifically against non-self nucleic acids. During the extensive course of systematic studies in clinically relevant model systems, several important factors responsible for NANPs’ immune recognition have been discovered [6], [7], [193], [194], [195], [196], [197], [198], [199], [200], [201], [202], [203]. For example, carrier molecules are required for the intracellular delivery of NANPs and their immunorecognition. In addition, the carrier provides extensive protection for NANPs from degradation by nucleases and defines NANPs biodistribution. Without any carriers, all tested NANPs have been effectively invisible to the cells of the immune system. Toll-like receptors in endosomal compartments are the first line of defense against NANPs that undergo scavenger receptor mediated endocytosis and there are various mechanisms with which the immune system responds to NANPs. The recognition of NANPs by human immune cells normally activates a complex network of signaling cascades with excreted interferons being produced as key cytokines. Upon the assessment of immunostimulatory properties of a representative set of NANPs, their dimensionality (1D, 2D, or 3D), composition (RNA vs DNA), and extend of functionalization were found to be the main contributors to the immunostimulation (Fig. 12).

Mechanistic studies helped to disseminate how NANPs can be recognized by the immune cell machinery to trigger the observed immune responses. For example, endosomal TLR7 and TLR9 and a cytosolic RIG-I have been identified to be important regulators of NANPs’ immune recognition by human immune cells [193], [194], [196], [204]. The chemical composition of NANPs also defines their interactions with PRRs as well as their localization within a cell. For example, while in the cytoplasm, some RNA made NANPs may be recognized by RIG-I and DNA hybrids with 2‘ fluorination can be recognized by RNA polymerase III that transcribes RNAs amenable for detection by RIG-I [196].

Overall, NANPs technology introduces a unique class of therapeutics that bring new possibilities for immunotherapies and targeted drug delivery with their structural versatilities. These particles have the potential to interact with human immune system in unique ways and, if designed correctly, can become a basis for a new class of safe and effective therapeutics. The knowledge and experience we gained by now are testimony to NANPs' potential. Further studies of how NANPs produce the observed immunological effects will pave the way for novel applications.

## 3 Summary of the third conference on biomotors, viral assembly and RNA nanotechnology

### 3.1 Meeting summary and logistics

The conference was successfully delivered over a period of four days, December 12–16, 2021, *via* the Zoom webinar platform. The first day of the conference presentations were focused on viral nanomachines and vaccines. Throughout these sessions, speakers presented their research on molecular machines and other platforms to understand viral mechanisms to produce better antiviral therapeutics including SARS- CoV-2. This day included a variety of computational and experimental results, highlighting the synergy between these two approaches to solving biochemical problems.

The second day of the conference was themed around RNA nanotechnology and therapeutics. Much of RNA’s unique structural and functional properties were exhibited here. There is a tremendous effort to understand and predict the structure and function of RNA in therapeutic applications using computational methods to model RNA platforms. One of the highest barriers to overcome in RNA therapeutics is delivery into the cytoplasm. Researchers presented current carrier options and the about the search for alternative methods. Applications of RNA nanotechnology were also discussed, in particular their uses in gene therapies, diagnostics, and targeted delivery were highlighted.

Day three looked into modeling nanostructures for health and life and engineering molecular machines to mimic motor proteins. There was also significant interest in nanozymes to perform enzyme like functions for therapeutics against cancers and viral infections. The design and function of anti-cancer RNA therapies were discussed at length, including methods for effective delivery and the uses of small-interfering RNAs. It was also shown how computational modeling helps to inform the understanding and design of RNA based platforms by improving our ability to predict RNA structures and interactions.

The final day of the conference focused more heavily on biomotor imaging, nanopore sensing and DNA packaging systems. The design of nanopores for various molecular functionalities, including sequencing, was discussed, with talks highlighting the ability of nanopores to function with both proteins and nucleic acids. Methods for using nanopores to monitor reaction kinetics were also shown. DNA delivery methods into cells were discussed in depth and attendees were shown how, by utilizing molecular machines, DNA can be packaged and excreted from viruses and virus-like particles. There was also substantial attention given to the structure, functions, and mechanisms of ATPase Motors.

Lewis Rolband, Damian Beasock, Yelixza Avila, and Leyla Danai (University of North Carolina at Charlotte) moderated the conference through the assignment of presentation permissions to speakers, enforcing time requirements, transitioning between sessions, introducing session moderators and keynote speakers, and assisting with any minor technical difficulties that arose during the event.

The core mission of the International Society of RNA Nanotechnology and Nanomedicine (ISRNN) is to promote the most recent headway in RNA nanotechnology while facilitating the exchange of information and formation of interdisciplinary collaborations among researchers around the world. Founded in 2016, the society focuses heavily on the applications of RNA nanotechnologies as RNA therapeutics, biomedicines, diagnostics, and imaging. The leadership team of the ISRNN is comprised of more than 30 distinguished researchers from seven countries, with members across the globe. In order to further their primary objectives of disseminating the latest research and connecting the researchers behind these advancements, the ISRNN hosts regular conferences. In addition to the Biomotors, Viral Assembly, and RNA Nanobiotechnology conference series, the ISRNN hosts other meetings, such as their 2020 Webinar Series, which had speakers and attendees from at least 10 countries. Members of the ISRNN are given priority to organize meeting programs, speak at all meetings hosted by the society, receive discount for publications at ISRNN sponsored journals, and have access to a variety of benefits such as free RNA Nanotechnology books and privileged publications.

The 3rd Biomotors, Viral Assembly, and RNA Nanobiotechnology conference was a resounding success that brought researchers together from across the globe through a convenient online only format. This format allowed for the conference to have a much greater reach than an in-person event would have, and, as such, it brought together as many researchers as possible in these and related fields to share highlights of their most recent work and catalyzed the discussion and development of new ideas. RNA has powerful therapeutic potential; by highlighting recent advances and connecting researchers, the ISRNN hopes that RNA nanotechnology and nanomedicines can reach their full potential as quickly as possible.

## Declaration of Competing Interest

The authors declare that they have no known competing financial interests or personal relationships that could have appeared to influence the work reported in this paper.
